# The Role of Latently Infected B Cells in CNS Autoimmunity

**DOI:** 10.3389/fimmu.2015.00544

**Published:** 2015-10-28

**Authors:** Ana Citlali Márquez, Marc Steven Horwitz

**Affiliations:** ^1^Department of Microbiology and Immunology, The University of British Columbia, Vancouver, BC, Canada

**Keywords:** multiple sclerosis, EBV, B cells, γHV-68, EAE

## Abstract

The onset of multiple sclerosis (MS) is caused by both genetic and environmental factors. Among the environmental factors, it is believed that previous infection with Epstein–Barr virus (EBV) may contribute in the development of MS. EBV has been associated with other autoimmune diseases, such as systemic lupus erythematous, and cancers like Burkitt’s lymphoma. EBV establishes a life-long latency in B cells with occasional reactivation of the virus throughout the individual’s life. The role played by B cells in MS pathology has been largely studied, yet is not clearly understood. In MS patients, Rituximab, a novel treatment that targets CD20^+^ B cells, has proven to have successful results in diminishing the number of relapses in remitting relapsing MS; however, the mechanism of how this drug acts has not been clearly established. In this review, we analyze the evidence of how B cells latently infected with EBV might be altering the immune system response and helping in the development of MS. We will also discuss how animal models, such as experimental autoimmune encephalomyelitis (EAE) and murine gammaherpesvirus-68 (γHV-68), can be used as powerful tools in the study of the relationship between EBV, MS, and B cells.

## Introduction: MS and Environmental Factors

MS is a neurodegenerative disease that affects the central nervous system (CNS). Largely accepted as an autoimmune disease, the mechanism of how MS develops is still not clear. However, thanks to studies using experimental autoimmune encephalomyelitis (EAE), the animal model for MS, we now know that MS lesions are caused primarily by myelin-specific T cells and macrophages that infiltrate the brain and cause myelin degradation and axonal degeneration (1). The primary T cells that infiltrate the CNS are CD4^+^, Th1, and Th17 cells. These cells initiate acute lesions that are characterized by the breakdown of the blood–brain barrier (BBB), which drives the inflammatory process of MS (2). In addition, CD8^+^ T cells that recognize myelin proteins can also can be found in the perivascular regions (3). These regions also contain other immune cells, such as dendritic cells (DCs), B cells, microglia, astrocytes, macrophages, and natural killer T cells (NKT) (4).

While the etiology of MS is still unknown, several genetic and environmental factors have been identified as possible elements that increase the risk of developing MS. Among the specific genetic markers related to the development of MS is the presence of genes related to alleles in the human leukocyte antigen (HLA) class II region [which is part of the major histocompatibility complex (MHC)], especially genes containing HLA-DRB1*15.01 (5, 6). While genome-wide association studies (GWAS) have identified several non-MHC associations with MS in Caucasian populations, these appear to have a modest impact in the overall risk of disease, making MHC the main susceptibility locus (6, 7).

In addition to genetic research, studies in migration, discordancy among identical twins, and geographical gradients strongly suggest that environmental factors influence susceptibility to MS. Several environmental factors have been linked to increased risks of developing MS, including vitamin D deficiency (8), cigarette smoking (9), and infection by viruses, such as Epstein–Barr virus (EBV) (10). Among these factors, the relationship between EBV and MS is one that provides the strongest evidence of association. Though studies involving MS patients, together with investigation using EAE and other animal models of MS have yielded high quantities of data, the extent of the contribution of environmental factors in the onset of autoimmunity is still widely unknown. In the following sections, we explore some of the proposed mechanisms for how previous infection with EBV can contribute to MS, discuss the importance of B cells on MS pathology, and finally, propose an animal model that will help to further explore the relationship between EBV, memory B cells, and the pathology of MS.

## Epstein–Barr Virus and Multiple Sclerosis

Epstein–Barr virus is a γ-herpesvirus that infects both epithelial cells and B cells (11). Infected B cells are activated and differentiate to memory B cells, which then are released to peripheral circulation where they are recognized by T lymphocytes (12). Although the immune system is able to control the EBV infection, the provirus remains latent in the host’s B-lymphocytes for the rest of his/her life. During latency, the main reservoir for EBV is long-lived memory B cells that have gone through somatic hypermutation and immunoglobulin class-switch recombination (13). The host cell expresses EBV gene products including six nuclear proteins (EBNA-1/2/3A/3B/3C/LP), three membrane proteins (LMP-1/2A/2B), and EBV-encoded small RNAs (EBER-1 and EBER-2). These products can control the host’s cell cycle and prevent apoptosis. The virus reactivates again at intervals during the host’s life (14). Primary infection with EBV is transmitted through saliva and, when it occurs during childhood, is asymptomatic (15). In contrast, if the infection occurs during puberty or early adulthood, it can cause infectious mononucleosis (IM), which is characterized by vague malaise followed by fever, sore throat, swollen posterior cervical lymph nodes, and fatigue (14).

Historically, EBV infection has been associated with the development of several autoimmune diseases and cancers. Some of these include Burkitt’s lymphoma, systemic lupus erythematosus (SLE), rheumatoid arthritis (RA), and MS.

A connection between MS and EBV was first suggested when it was recognized that there are similarities in the demographic distribution of MS and IM (10), whereby both IM and MS occur at higher incidences in developed countries. Subsequent studies found that although 90% of the general population has circulating anti-EBV antibodies, these antibodies are found in almost 100% of MS patients (16), and that people with a history of IM have a two to three times higher risk of developing MS (17). Contrastingly, in developing countries, where infection with EBV occurs early in life, individuals show a low incidence of IM and, consequently, the risk of developing MS is much lower (18, 19). This so-called “paradox” reveals that the relationship between MS and EBV is related to the stage in life when the infection with EBV occurs (16), together with the associated development, or not, of IM. The factors that determine the relationship between EBV, IM, and MS, however, have not yet been clearly established.

In support of the epidemiological data, it has been described that MS patients show increased levels of serum or plasma IgG antibodies against the EBNA family in general, and in particular against EBNA 1, EBNA2 (20), EBNA3 (EBNA3A), EBNA4 (EBNA3B), EBNA6 (EBNA3C), LMP1, EBV capsid protein VP26 (21), early antigen complex (20, 22) EBV viral capsid antigen (23), and the EBV lytic protein BRRF2 (24). In addition, patients with MS also have elevated levels of these antibodies in the cerebrospinal fluid (CSF), including IgG antibodies to EBNA1, viral capsid antigen, EBV early antigen, Epstein–Barr virions and BRRF2 (24). Furthermore, increased antibody titers have been observed in adults more than 10 years before the development of the first MS symptoms (25).

Several hypotheses have been proposed to explain the relationship between EBV and MS. Among these, the most studied are molecular mimicry, bystander damage and mistaken self, and the EBV-infected autoreactive B cell hypothesis.

### Molecular Mimicry

This hypothesis postulates that T cells specific for EBV antigens (such as EBNA-1) are structurally related to CNS antigens like myelin basic protein (MBP). In this way, a TCR would be able to recognize more than one peptide and lead to recognition of autoantigens (26, 27). Additionally, it has been shown that anti-EBV antibodies, such as anti-EBNA-1, are cross-reactive for epitopes of neuroglial cells (28) and transaldolase, a protein expressed selectively in oligodendrocytes (29). Although this theory explains the development of autoreactive immune cells, it is not likely to be the sole cause of the onset of the disease, as the development of autoreactive cells and antibodies still requires leakage past the BBB and some targeting or inflammation at site of damage. Further, the presence of latently infected B cells alone does not necessarily influence cross-reactivity. Though the presence of latently infected B cells in the brains of MS patients (30) remains controversial (31, 32), B cells and plasma cells are commonly found in MS lesions, appear in large numbers in chronic MS plaques, and are present in areas of active myelin breakdown (33). Moreover, lymphoid B cell follicle-like structures that feature characteristics similar to germinal centers have been observed in the cerebral meninges of MS patients with secondary progressive MS and are usually associated with cortical neuronal loss and demyelination (34).

### The Bystander Damage Hypothesis

This hypothesis establishes that the activation of CD8^+^ or CD4^+^ T cells directed against EBV antigens, particularly lytic antigens, can result in bystander damage to the CNS. However, in order for this hypothesis to be possible, it would be necessary for infected B cells to be present in the CNS, which has been rather hard to prove. Serafini et al. showed that meningeal B cell follicles and acute white matter lesions express EBV nuclear transcripts (EBERs) (30); however, further attempts to detect EBV in MS brains have been futile (31, 32, 35). Under the bystander damage hypothesis, MS would not be an autoimmune disease, *although* secondary autoimmune responses could occur as a result of sensitization to CNS antigens released after virus-targeted bystander damage (30). A caveat to this hypothesis would be that overall and relative to other viruses, EBV does not directly damage the cells that it infects, leaves little bystander inflammation and is not likely to induce disease through this type of mechanism preferentially in the CNS.

### Mistaken Self Hypothesis

In this hypothesis, the stress protein αB-crystallin that is expressed *de novo* in infected lymphoid cells is recognized by T-cells that are activated by microbial antigens, hence the accumulation of the αB-crystallin self antigen in oligodendrocytes provokes a CD4 T cell response with resultant demyelination (36). To date, little to no data exists to fully support this scenario.

### The EBV-Infected Autoreactive B Cell Hypothesis

Pender has proposed a new theory, where EBV specific CD8^+^ T cells do not effectively eliminate EBV-infected B cells, leading to the accumulation of autoreactive B cells infected with EBV in the CNS (37). If this theory proves true, it is possible that boosting the immune system with CD8^+^ T cells specific for EBV epitopes could be a successful treatment for MS patients.

In support of this theory, Pender et al. recently performed a trial where they treated a patient with secondary progressive MS using AdE-1-LMPpoly, a recombinant adenovirus vector that encodes multiple CD8^+^ T-cell epitopes from the latent EBV proteins EBNA1, LMP1, and LMP2A (38). The patient was treated with EBV specific CD8 T cells expanded with AdE-1-LMPpoly and IL-2. The results showed an improvement in symptoms including reduction in fatigue and pain. More studies are needed in order to determine if this regimen could be effective in treating secondary progressive MS. In addition, more research is needed to investigate the treatment’s mechanism of action, which is believed to occur through the elimination of EBV-infected B cells in the CNS (39). Nonetheless, since this treatment depletes B cells in general, it may block a number of putative autoimmune mechanisms and does not specifically demonstrate Pender’s hypothesis.

In summary, these four hypotheses explain some of the potential scenarios that contribute to the development of autoimmunity by EBV. However, each of them fails to explain key characteristics of MS pathogenesis. Since sample collection from MS patients is limited, the development of animal models to help understand and explain these hypotheses is imperative and will eventually help us to understand the role of latently infected B cells in this relationship.

**Figure 1:**
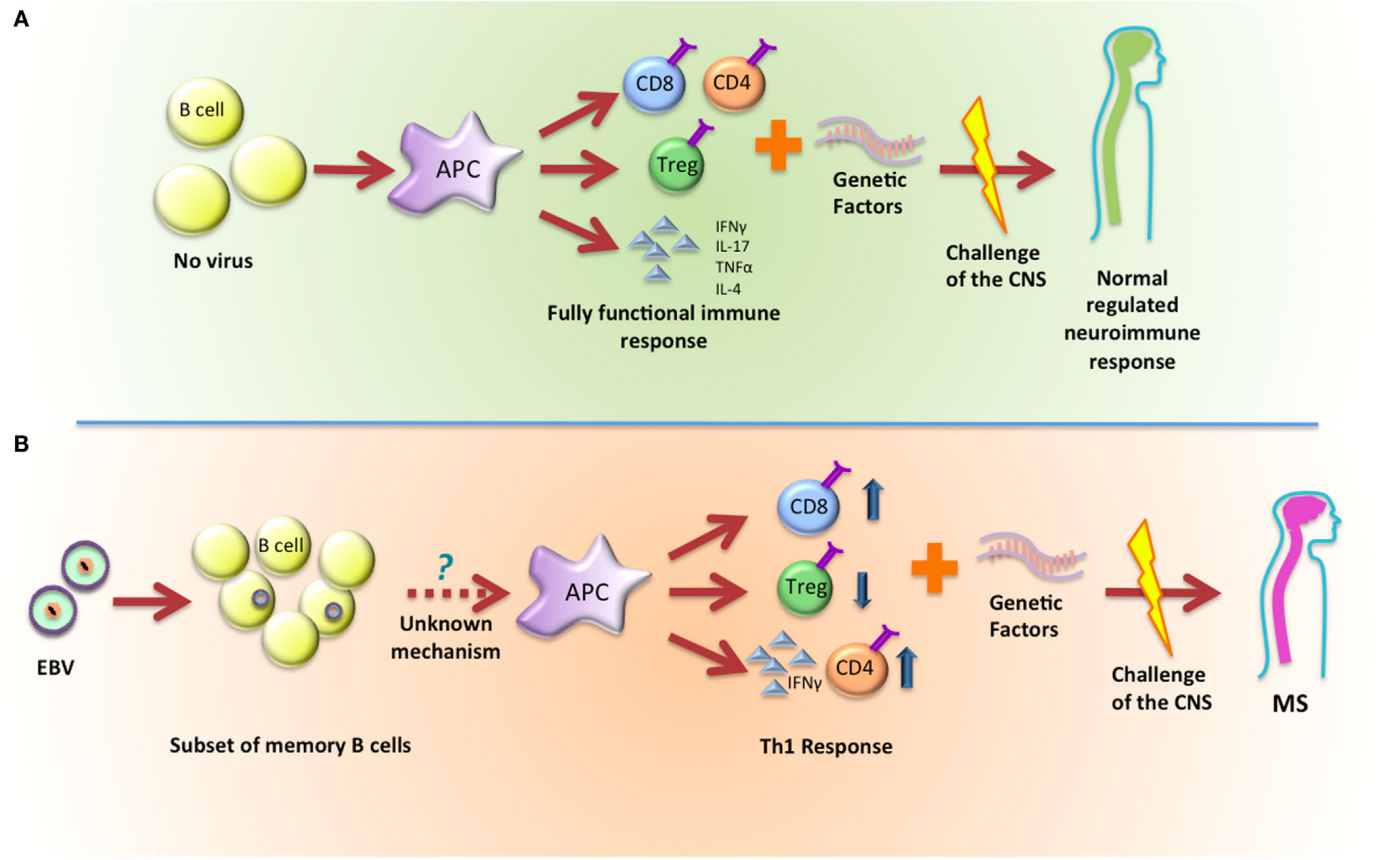
**Latent infection with EBV establishes a precondition that leads to the development of MS**. **(A)** In normal conditions, B cells will activate APCs and, depending on the stimulus they receive, they can promote a fully functional immune response that will not impact the development of MS. **(B)** EBV latently infected B cells will activate APCs and promote a skewed Th1 response that, when combined with genetic factors, will lead to the development of MS.

As an alternative to these hypotheses, we propose that EBV infection and latency establishes a precondition to the immune response where subsequent challenges show acceleration and/or enhanced Th1 outcomes that eventually will lead to the onset of MS (Figure 1). In this scenario, the latently infected B cell is not an initiator but instead acts as a necessary co-factor in disease progression.

## The Importance of B Cells in Multiple Sclerosis

B cells found in the CNS and CSF of MS patients are clonally expanded and have gone through IgG class-switch and somatic hypermutation (40–42). In MS patients, more than 90% of B cells in the CSF express the memory B cell marker CD27 and a fraction of CSF B cells express CD138 and/or CD38, suggesting stimulation of the maturation of clonal activated memory B cells into antibody producing plasma blast. On the other hand, naïve B cells expressing CD27 IgD^+^ naive B cells are significantly lower in the CSF compared to blood (43, 44). The memory B cells that can be found in the CSF have an upregulation of co-stimulatory molecules, which suggests an active B and T cell interaction (45).

Until recently, it was believed that the only role B cells played in MS pathogenesis was the production of autoantibodies; however, with the realization that B cell depleting drugs, such as Rituximab, ocrelizumab, and ofatumumab, had an important effect in diminishing relapses in patients with relapsing-remitting MS (RRMS), it has became more evident that B cells may be acting as antigen-presenting cells (APCs) during MS. In fact, patients treated with B cell depleted therapy show a rapid response to the treatment, and since these antibodies do not affect plasma cells, it is now believed that autoantibodies are not as important in the pathogenesis of MS as B cells functioning as APCs or immunomodulators (46–49). In their role as APCs, it has been suggested that B cells and DCs interact via cytokine-dependent feedback loops to shape the T cell response to viral infections. When B cells are stimulated with cytokines, TLR ligands, or antibodies, these cells release diverse cytokines including IL-10, TGFβ, IL-6, or IL-17, which have a suggested modulatory effect in DCs (50–52). One of these effects is the suppression of Ag presentation by IL-10. It has also been seen that high levels of TGFβ are produced by B cells stimulated with LPS, which regulates Th1 response in NOD mice, induces the apoptosis of T cells, and impairs the ability of APCs to present auto-Ags. In addition, IL-6 promotes the differentiation of B cells into Ab secreting plasma cells in mice and humans, and IL-17 has been seen to control DC maturation in mice infected with *Trypanosoma cruzi* (53). Alternatively, IL-12 production on DCs inhibits T cell derived IFNγ, as well as the production of pro inflammatory cytokines through its actions on DCs (54).

In autoimmunity, the APC role of B cells has been primarily studied in EAE, which has long been accepted as the best *in vivo* model of MS. In active EAE, mice are immunized with myelin peptides, most often derived from either MBP or myelin oligodendrocyte glycoprotein (MOG) that are emulsified in complete Freund’s adjuvant (CFA, which is composed of mineral oil and desiccated *Mycobacterium tuberculosis*) (55). In addition, two injections of pertussis toxin (PTX) might be needed, depending on the strain of mouse used. EAE leads to an ascending paralysis in 10–12 days after induction and is characterized by a CD4-mediated autoimmune reaction. SJL mice injected with PLP generally develop a relapsing-remitting disease course. In C57Bl/6 mice, EAE induction with MOG results in a chronic progressive disease (55). Alternatively, passive EAE can be induced if MOG-specific T cells are transferred to naïve mice.

It has been observed that B cell antigen presentation plays a critical role in the initiation of EAE (56, 57). Mice with a BCR specific for MOG but that cannot secrete antibodies are susceptible to EAE, while mice deficient in MHC II on B cells are resistant to EAE (58). This is further confirmed in patients since it has been described that contrary to other autoimmune diseases, such as RA, central tolerance of B cells is not affected in MS. Instead, only peripheral tolerance seems to be defective in MS, which can be the result of defective Treg function (59, 60). Patients with RRMS show memory B cells in peripheral blood, which are able to respond to MBP. Finally, it has been described in patients with MS that a pool of IgG-expressing B cells is capable of bidirectional exchange through the BBB (7).

One of the important characteristics of B cells in MS patients is cytokine production. In EAE mice, B cell depletion seems to deplete B cells that are producing IL-6, which helps to ameliorate symptoms of the disease. In MS patients, B cells produce higher levels of IL-6 compared to healthy controls. After depletion of B cells with anti-CD20, and after B cell reconstitution, the new cells do not seem to produce the same level of IL-6 than before depletion, which might help to understand the ameliorating effect in patients. All of this is accompanied by reduced levels of IL-17 secreted by peripheral T cells (61). In contrast, B cells that show a regulatory phenotype, commonly referred as Bregs or B10 cells, due to their ability to secrete IL-10, a cytokine known to be immunoregulatory, are able to modulate the autoimmune response in EAE (62). In RRMS, it has been shown that during relapses, patients have reduced levels of Bregs as well as memory B cells in peripheral blood compared to healthy donors (63, 64).

## Animal Models to Study Multiple Sclerosis and Epstein–Barr Virus Infection

While numerous lines of evidence point toward a relationship between MS and EBV, the study of this interaction is limited since EBV only infects humans and, while most patients become infected with the virus during childhood or adolescents, the onset of MS does not occur until years later.

Despite these limitations, a current murine virus can be used to study γ-herpesviruses. Murine γ-herpesvirus 68 (γHV-68), is a γ-herpesvirus that has provided a widely used model to study human γ-herpesviruses, in particular EBV and Kaposi’s sarcoma-associated herpesvirus (KSHV) (65). γHV-68 shares most of its genomes with these two viruses, and, importantly, genes that are associated with EBV cell tropism – latency and transformation – are present in γHV-68 (66).

Mice are inoculated with γHV-68 usually via intraperitoneal or intranasal methods. Despite of the route of infection, the virus main reservoir will be the spleen and it will be cleared 14–16 days post infection, at that point, it will establish a life-long latency in primarily isotype-switched B cells CD19^+^ IgD^−^, which are considered as memory B cells (67). During early stages of latency, the virus also establishes itself in macrophages and splenic DCs, although to a much lesser extent. In these other APCs, γHV-68 latency decreases considerably with time (68, 69).

In γHV-68 infection, the virus is able to modify the expression of different genes in the cells that harbor the latent virus. Many of these genes are inflammatory cytokines, such as IFNγ, IL-18 receptor, SOCS3, and a wide array of known stimulated IFNαβ genes (50–52). But also, once the virus has established latency in B cells, it continues expressing latency genes that are able to regulate the expression of genes in B cells. In the same way, B cells will express other genes in order to control γHV-68 reactivation. All of this will bring different outcomes that will differentiate latently infected B cells from uninfected B cells.

Among the viral genes expressed during latency, we can find M2, a protein that can suppress STAT1/2 expression and that as a consequence, leads to the inhibition of the interferon response (70), as well as being able to induce the expression of IL-10 in primary B cells. Despite M2 being unique for γHV-68, EBV also is able to modulate the immune response by producing its own viral IL-10 (vIL-10) (71). In addition, M1 a secreted protein with a superantigen-like activity might play an important role in maintaining latency (72). Additionally, EBV encodes for 25 pre-miRNAs that may play a role in immune response whose target transcripts are immune recognition, apoptosis, and cell cycle pathways. γHV68 can generate 15 mature miRNAs; however, their function is less understood than in EBV (73, 74). However, it is known that micro RNAs are not necessary for acute replication, but that they are important in the establishment of latency in germinal center and memory B cells (75).

Infection of γHV-68 is able to increase Heparin sulfate (HS) in the surface of B cells. HS is a co-factor for cytokines, chemokines, and growth factors, and its upregulation is dependent on the expression of type I IFNs that increase responsiveness to APRIL, a cytokine important for B cell survival and T cell-independent B cell responses (76). It is well known that IFN α/β are important to direct γHV-68 into latency, and that they are also important in maintaining latency (77). Moreover, Latency Membrane Protein (LMP-1) is a virus protein that has been shown to control EBV’s latent life cycle. LMP-1 is upregulated in the presence of Type I IFN, in particular IFN α (78), and this unique feedback maintains the latent life cycle and as well as promotes host IFN production (79). Intriguingly, it is important to remark that IFN α and IFN β present functional differences (80) that are in a unique balance with each other. While not completely understood, Type I IFNs have been largely used in the clinic with different purposes, while IFN α is used to treat chronic hepatitis C infection, IFN β has been effective for the treatment of MS. Addition of either IFN α or IFN β generally resolves in diminishment of the other. Based on the effectiveness of Betaferon in the clinic and its putative role in upsetting the balance between LMP-1 and IFN α, a better understanding of the roles and functions of IFN α and IFN β should be explored in the context of EBV infection and MS. In particular, it would be interesting to explore whether IFN α/β produced by infected B cells for the maintenance of latency are able to promote APC maturation.

There is even stronger evidence that γHV-68 a successful model to help understand the relationship between EBV and MS. Peacock et al. describe that EAE induced mice infected with γHV-68 show exacerbated symptoms of EAE compared to non-infected mice (81). Moreover, similar to what is observed in MS patients, it has been described that γHV-68 is capable of inducing the expression of αB crystallin in mice infected with the virus. These mice develop a strong immune response against heat shock protein (82). These experiments, however, do not address the changes in the pathology of EAE or MS.

Combining EAE and γHV-68 models, our research has focused on determining the relationship between EBV infection and the onset of MS. Recently, we demonstrated that mice that were latently infected with γHV-68 before the induction of EAE showed increased ascending paralysis, as well as augmented neurological symptoms and brain inflammation. This was the result of a stronger Th1 response in infected mice, characterized by higher levels of IFNγ and diminished IL-17 levels. CD8 infiltration into the CNS was also noted in these latently infected mice. This is remarkable, given that EAE pathology generally lacks the presence of CD8 T cell infiltration and has a predominant Th17 response. Conversely in MS, CD8 T cells infiltration and a combined Th1/Th17 response are hallmarks of disease pathology. Another important aspect is the upregulation of the co-stimulatory molecule CD40 on APCs during EAE induction in latently infected mice (83). Recently, we showed that the enhanced disease observed in γHV-68 latently infected mice depends on maintaining the latent life cycle of the virus, and this is strongly associated with pSTAT1 and CD40 upregulation on uninfected CD11b^+^CD11c^+^ cells. This CD40 upregulation leads to a decrease in the frequency of regulatory T cell (84). CD40 signaling is important in the activation and suppression of Tregs and that its upregulation is associated with an enhanced Th1 response and fewer Tregs. Further, it has been associated with the development of autoimmunity (85, 86). Moreover, the decrease in peripheral Treg frequencies observed in latent γHV-68 infection is also well described in MS patients (87, 88). It is highly likely that the mechanisms in place that maintain latency also modulate a pro-Th1 response and reduce Treg control. This results in prevention of virus reactivation and may not always be in the best interest of the virus. IFN α/β are required for the maintenance of latency and are likely candidates for the Th1 modulation. Further research is needed to determine if factors, such as IFN α/β, are involved in the enhancement of EAE symptoms, and in particular, to understand potential differences between uninfected and latently infected B cells.

Finally, studies performed on non-human primates would be an important tool in the study of EBV and MS. In marmosets, for example, EAE is effectively inhibited when marmosets are treated with anti-CD20; however, treatment with anti-BlyS or anti-APRIL, which mainly depletes peripheral B cells, but not CD40^high^ B cells, only delays the onset of EAE (89, 90). It has been proposed that the difference in the effectiveness of the treatments resides in the fact that cells infected with CalHV3 are among the B cells depleted by anti-CD20; CalHV3 is the marmoset equivalent of human EBV, and is a B-cell transforming lymphocryptovirus (91). Moreover, it has been described that a small percentage of Japanese macaques which are naturally infected with a gamma 2-herpesvirus, named JM radhinovirus, isolated from CNS lesions, spontaneously develop an encephalomyelitis that is similar to MS (92). In addition, since EBV has not just been associated with MS but with other autoimmune diseases like lupus and inflammatory bowel disease, it is possible that the mechanism of action is similar in these diseases, making γHV-68 even more important in the study of the development of autoimmunity.

It is our contention that EBV acts a co-factor that sets up a precondition in which any subsequent environmental stress runs the risk of an overly responsive, under regulated Th1 response. Specificity toward the CNS, myelin sheath, and oligodendrocyte is dictated by the secondary stress event and not EBV latency. While EAE is an acceptable model that mimics many of the characteristics of MS, it does not represent how MS is induced; given that not every person infected with EBV develops MS, genetic predispositions, as well as other environmental factors must be involved in the expression of the disease. With that in mind, other environmental events and stresses that target the myelin sheath or oligodendrocytes, such as a secondary virus infection or toxin, likely act to initiate the disease in the presence of latently infected B cells. For example, agents like cuprizone, a copper-chelating agent, that is known to cause demyelination in the CNS through oligodendrocyte apoptosis (93, 94), may well be active MS inducers.

By studying these models in the context of latently infected B cells, we will be able to better investigate the role of latent virus infection in the initiation and progression of MS.

## Conclusion

Determining the mechanism that describes how environmental factors, such as EBV and IM, are related to the onset and development of MS is vital to understanding how MS pathogenesis is developed. The efficacy of treatments, such as Rituximab and Betaferon that indirectly act to inhibit EBV latency in B cells by depleting B cells or upsetting the IFN balance, serves to demonstrate the important role that EBV latent infection plays in MS progression. It is also important to remember that neither B cell depletion nor IFN I addition are successful therapies for EAE and were instead chosen because of their efficacy in other autoimmune diseases. With the aid of new animal models that consider the role of latent infection, it is expected that these complicated causal mechanisms can be more easily studied and new and more effective treatments for MS patients will more closely at hand.

## Conflict of Interest Statement

The authors declare that the research was conducted in the absence of any commercial or financial relationships that could be construed as a potential conflict of interest.
